# Estimation of the serial interval and proportion of pre-symptomatic transmission events of COVID− 19 in Ireland using contact tracing data

**DOI:** 10.1186/s12889-021-10868-9

**Published:** 2021-04-27

**Authors:** Conor G. McAloon, Patrick Wall, John Griffin, Miriam Casey, Ann Barber, Mary Codd, Eamonn Gormley, Francis Butler, Locksley L. McV Messam, Cathal Walsh, Conor Teljeur, Breda Smyth, Philip Nolan, Martin J. Green, Luke O’Grady, Kieran Culhane, Claire Buckley, Ciara Carroll, Sarah Doyle, Jennifer Martin, Simon J. More

**Affiliations:** 1 School of Veterinary Medicine, University College Dublin, Belfield, Dublin 4, Ireland; 2 School of Public Health, Physiotherapy and Sports Science, University College Dublin, Belfield, Dublin 4, Ireland; 3 Centre for Veterinary Epidemiology and Risk Analysis, School of Veterinary Medicine, University College Dublin, Belfield, Dublin, Ireland; 4 School of Biosystems and Food Engineering, University College Dublin, Belfield, Dublin 4, Ireland; 5 Department of Mathematics and Statistics, University of Limerick, Limerick, Ireland; 6 Health Information and Quality Authority, George’s Court, Dublin 7, Ireland; 7 Department of Public Health, Health Service Executive West, Galway, Ireland; 8 National University of Ireland Maynooth, Kildare, Ireland; 9 School of Veterinary Medicine and Science, University of Nottingham, Nottingham, UK; 10 Central Statistics Office, Ardee Road, Rathmines, Dublin, Ireland; 11 COVID-19 Contact Management Programme, Health Service Executive, Dublin, Ireland; 12 School of Public Health, University College Cork, Cork, Ireland

**Keywords:** COVID-19, SARS-CoV-2, Contact tracing, Serial interval

## Abstract

**Background:**

The serial interval is the period of time between the onset of symptoms in an infector and an infectee and is an important parameter which can impact on the estimation of the reproduction number. Whilst several parameters influencing infection transmission are expected to be consistent across populations, the serial interval can vary across and within populations over time. Therefore, local estimates are preferable for use in epidemiological models developed at a regional level. We used data collected as part of the national contact tracing process in Ireland to estimate the serial interval of SARS-CoV-2 infection in the Irish population, and to estimate the proportion of transmission events that occurred prior to the onset of symptoms.

**Results:**

After data cleaning, the final dataset consisted of 471 infected close contacts from 471 primary cases. The median serial interval was 4 days, mean serial interval was 4.0 (95% confidence intervals 3.7, 4.3) days, whilst the 25th and 75th percentiles were 2 and 6 days respectively. We found that intervals were lower when the primary or secondary case were in the older age cohort (greater than 64 years). Simulating from an incubation period distribution from international literature, we estimated that 67% of transmission events had greater than 50% probability of occurring prior to the onset of symptoms in the infector.

**Conclusions:**

Whilst our analysis was based on a large sample size, data were collected for the primary purpose of interrupting transmission chains. Similar to other studies estimating the serial interval, our analysis is restricted to transmission pairs where the infector is known with some degree of certainty. Such pairs may represent more intense contacts with infected individuals than might occur in the overall population. It is therefore possible that our analysis is biased towards shorter serial intervals than the overall population.

**Supplementary Information:**

The online version contains supplementary material available at 10.1186/s12889-021-10868-9.

## Background

COVID-19 was declared a pandemic on March 11, 2020 [1]. Epidemiological models of SARS-CoV-2 infection have been developed in many countries as aids to national-level decision making [2–4]. One use of such models is in the estimation of the reproduction number. The basic reproduction number, R_0_, is an indicator of the transmissibility of an infectious agent, defined as the expected number of new infections that are generated by a single infected individual over the course of its infectious period, in an otherwise susceptible population [5]. With the implementation of tiered control strategies, the number of secondary cases from an infected individual is expected to vary at particular points in time. The estimation of a time-varying reproduction number (Rt) allows the efficiency of infection transmission to be traced over time [6], providing some measure of the efficacy of control measures that have been introduced [7], as well as facilitating short-, and long-term predictions in case numbers.

One of the key parameters involved in estimating Rt from case data is the generation time (also called the generation interval), defined as the duration of time between two successive linked infection events, in other words the time between the point of infection for the infector and the point of infection for the infectee [8]. Since the precise time of infection is generally unobserved and therefore difficult to ascertain, the serial interval is often used as an approximate value for the generation time [9]. In contrast to the generation time, the serial interval is the duration of time between the onset of symptoms in the infector and the onset of symptoms in the infectee.

The serial interval is determined by a number of important factors: the contact patterns between infectious and susceptible individuals; the latent period and the duration of infectiousness; and the incubation period. The incubation period of COVID-19 is likely to be similar across populations and within populations over time [10]. Similarly, there does not appear to be any evidence to suggest that latent periods and duration of infectiousness are likely to vary across different countries [11]. In contrast, contact patterns are likely to be relatively specific to a particular population, therefore local estimates (for example at a national level) of the serial interval are preferable in the estimation of Rt [9, 12]. Traditionally, at least for the purpose of modelling, the serial interval is assumed to be a fixed duration that does not change within a population [12]. However, in the context of COVID-19, interventions have been introduced at different time points to reduce transmission by influencing human behaviour including general public health advice, regulations restricting work and social events, and movement restrictions. Consequently, the contact patterns of individuals are likely to change over time [13], resulting in temporal changes in the serial interval that can impact on the accuracy of the Rt estimate [12].

Similar to other countries, a national contact tracing service was established in Ireland to reduce transmission of infection. The primary goal was to instruct cases to self-isolate, to identify and provide advice to their close contacts to interrupt onward transmission, and to enable targeted testing of these close contacts. In addition, information was also gathered on the likely source of infection following discussion with that infected individual. This included reporting interactions with confirmed cases and clusters of cases reporting attending the same events and venues. Whilst these data were collected for a specific purpose, that is reducing onward transmission, secondary analysis of these data could be used to inform national controls.

The aims of the current study were:

1. To use contact tracing data to estimate the serial interval of SARS-CoV-2 in Ireland and,
2. To use the resulting serial interval distribution to estimate, using simulation, the proportion of transmission events that occurred prior to the onset of symptoms in Ireland.

## Materials and methods

### Description of the data

Data captured from the contact tracing process is described in detail elsewhere [13]. Briefly, details on cases and their contacts were collected during two phone calls which were held in two separate datasets (Dataset 1 and Dataset 2). The first call informed cases of their positive test status (or confirmed the test result if the individual had already been informed by their GP), collected the date of symptom onset (if any), as well as categorising the likely source of infection for that individual (close contact with a confirmed case, healthcare setting or community transmission - if the source was unknown). The second call collected details of the contacts of each confirmed case.

These data, based on data entries from contact tracing call centres, were collected by the Health Service Executive (HSE) under the Medical Officer of Health legislation, collected by the Central Statistics Office (CSO) in compliance with the Statistics Act 1993, pseudonymised, and stored in a centralised database (the CSO C19 Data Research Hub). The CSO C19 Data Research Hub is a secure data repository from which personally identifiable data cannot be exported.

These data were accessed through the CSO data hub by the first author for the purpose of this analysis. Access was granted under Section 20(b) of the Statistics Act, 1993, for the purpose of using data collected during the pandemic to aid in the national response. The study was approved by the National Research Ethics Committee (20-NREC-COV-099). The requirement for informed consent was waived by review board Health Research Declaration Committee (20–025-AF1/COV). All methods were carried out in accordance with relevant guidelines and regulations.

### Data linking/management

Contacts were linked to the primary case by joining Datasets 1 and 2 on the basis of the reference ID of the primary case. Next, contacts who subsequently became cases were identified by searching for the reference ID of the contact within the case database. To remove ‘mirror image’ transmission events (that is, those situations where a single transmission event is identified twice in the dataset, for example, one in which person A is the primary case, and person B is the secondary case; with a second entry where person B is the primary case and person A is the secondary case), we assigned a ‘pair id’ to each transmission event and retained only unique transmission events within the dataset.

### Study design

In order to restrict the analysis to definite infector-infectee pairs, data filtering processes were undertaken. Figures S1 and S2 (Supplementary Material) outlines potential errors that may arise, leading to incorrect specification of the infector for each infectee. To avoid the misidentification of an intermediate case in a close co-contact (Figure S1, Supplementary Material; secondary or tertiary case), we restricted our analysis to primary cases who only infected a restricted number of individuals: In the first instance, we restricted our analysis to transmission pairs where the primary case infected only one other individual. To evaluate the impact of this decision, we varied the cut-off point for the number of secondary cases per primary case from 2 secondary cases to 13 (maximum) and repeated the analysis. Whilst conducting these analyses in cases where the primary case resulted in more than one serial interval, we randomly sampled one of these for our analysis in order to account for the non-independence between multiple serial intervals from a single case.

To avoid the possibility that both the observed primary and secondary cases acquired the infection from a single unidentified infector (Figure S2, Supplementary Material; secondary case or common source), we restricted the analysis to cases where the most likely source of infection reported for the primary case was community transmission (that is, no source identified), whilst the source of infection reported for the secondary case was contact with a confirmed case. To reduce the potential for recall bias, we only used transmission pairs where contact tracing took place within 1 week of the onset of symptoms for both the primary case and secondary case. Finally, data were right censored to a point 30 days prior to the end of record collection to avoid bias from omitting longer serial intervals for which the date of symptom onset for any secondary cases had not yet been collected.

### Data analysis

The difference in time between the onset of symptoms between linked cases was calculated. Intervals greater than 28 days were removed from the dataset. This duration was chosen to correspond to the maximum possible duration of the serial interval given a maximum post-symptom onset infectious period of 13.4 days [11] and the 97.5th percentile of the incubation period [10]. Previous work has demonstrated that a small proportion of serial intervals are expected to be less than zero, in other words where the infectee displays symptoms before the infector [9]. Therefore we retained negative serial intervals in the dataset, but removed those less than − 10 days, the minimum serial interval reported by Du et al. [14].

Range, median, mean and interquartile range were summarised for the overall dataset, by age cohort of the primary case, age cohort of the infected contact, whether the primary case occurred in Dublin (the largest city) or the rest of the country and the restriction period during which the primary case occurred.

A range of statistical distributions were fit to the data: Weibull, gamma, normal and lognormal. These distributions were chosen as the distribution of serial intervals was expected to be positively skewed and they were consistent with the distributions that were used in previous studies of serial interval of COVID-19 [9]. Since the positively skewed distributions that we used are bounded by zero, and because of the possibility of negative serial intervals, a constant (k = 10 days) was added to each serial interval before fitting the distribution. The value for k was chosen to be sufficiently large such that its addition to each serial interval would equal a positive integer. Previous work has shown that a considerable proportion of serial intervals are negative, with a range of − 10 to 20 days [14]. The best fitting distribution was used as the final estimate of the serial interval distribution. Fit was determined in a number of ways: by the comparing lowest AIC; by plotting and comparing the Probability Density Function of each fitted distribution with the histogram of the raw data, and by plotting and comparing the Cumulative Distribution Function (CDF) of each fitted distribution against the Empirical Cumulative Distribution Function (ECDF) of the raw data. In addition, for each distribution we calculated the mean of the absolute difference between the of the raw data and the Cumulative Distribution Function (CDF) of the fitted distribution at each time point within the range of serial intervals in the data.

We simulated from the resulting distribution to estimate the proportion of transmission events that were likely to have occurred prior to the onset of symptoms of the infector. First, 100,000 random samples were drawn from the serial interval distribution, and k was subtracted from each sample. For each serial interval observation, we simulated 10,000 incubation periods using a lognormal distribution mu = 1.63 and sigma = 0.50 [10] and calculated the probability of pre-symptomatic transmission as the proportion of simulations where the serial interval minus the incubation period was < 0 (Supplementary Material Figure S3). The proportion of serial intervals with a probability of pre-symptomatic transmission > 0.5 was used as the estimate of the proportion of pre-symptomatic transmission in the population.

All data manipulation was conducted in R version 3.3.1 [15]. Parametric distributions were fit using the ‘fitdistrplus’ package in R [16].

## Results

### Descriptive statistics

Following initial data read in and selection of records with a primary case and contact identifier, there were 293,597 close contacts recorded from 111,251 unique primary cases. Data cleaning steps are detailed in Supplementary Material Table S1. The final dataset for analysis included 433 infected close contacts from 433 primary cases, from 11th April to 13th December 2020.

**Fig. 1:**
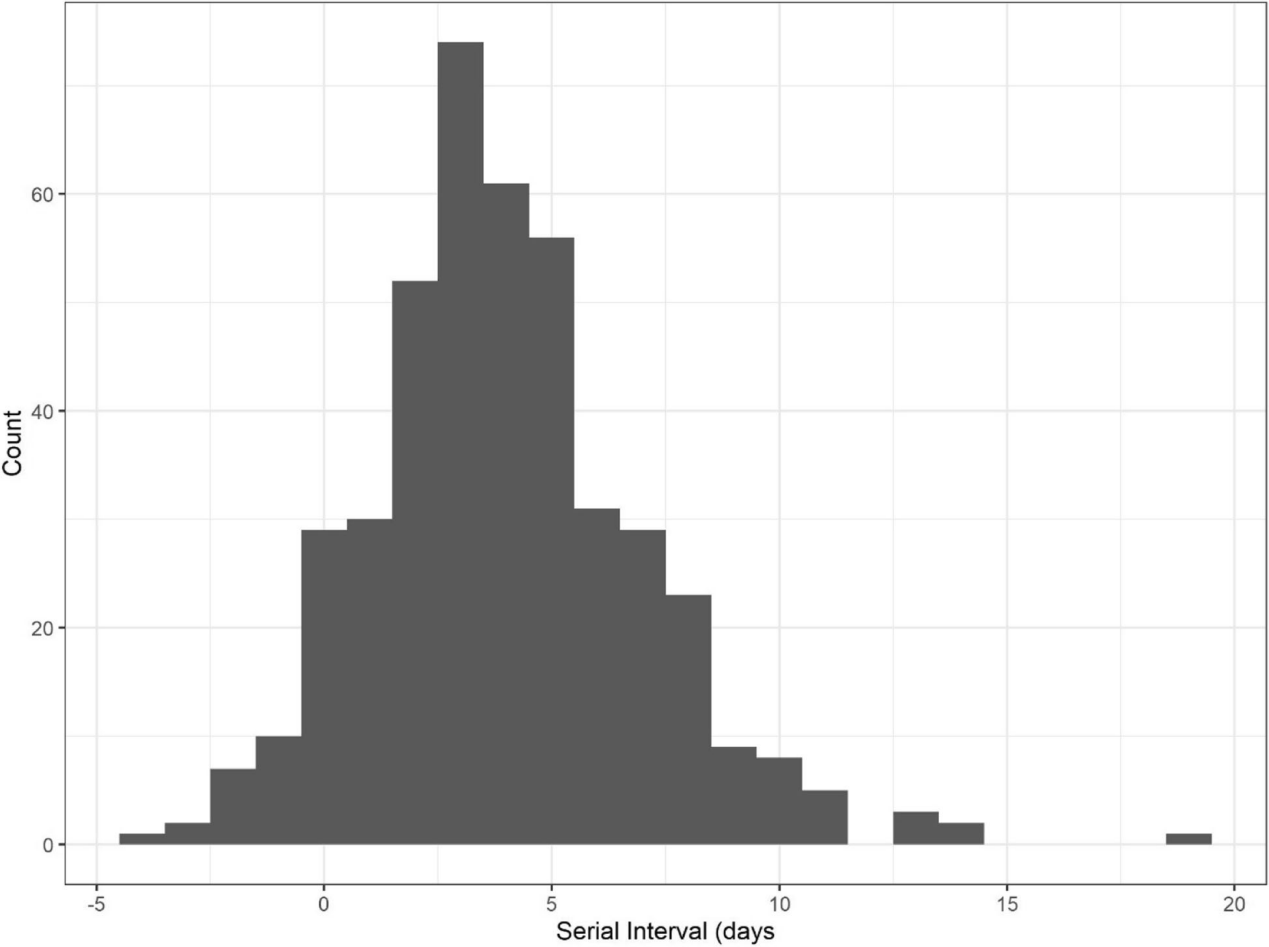
Histogram of serial intervals of transmission pairs infected with SARS-CoV-2 in the Republic of Ireland

Figure 1 shows the distribution of serial intervals. The median interval was 4 days, the 25th and 75th percentiles were 2 and 6 days respectively, mean interval was 4.0 days (95% confidence intervals 3.7, 4.3) and 4% of serial intervals were less than zero. Table 1 shows the breakdown of serial interval by location (Dublin versus the rest of the country), age cohort of the primary case, age cohort of the infected contact and level of restriction. When evaluated by age, serial interval was shortest when the primary case or the secondary case were in the older age cohort (≥65 years). Serial interval was similar across all restriction level time periods.

**Table 1:** Serial interval by location, age of primary and secondary case and restriction level

	Median Serial Interval	Mean Serial Interval (95% confidence intervals)	Standard deviation	Interquartile Range	Number of transmission events
Location					
Dublin	4	3.9 (3.4, 4.4)	3.0	3	137
Rest of Country	4	4.0 (3.7, 4.4)	3.0	4	296
Age of primary case					
0–18	4	3.4 (1.7, 5.1)	3.6	4	17
18–25	4	4.4 (3.7, 5.1)	2.9	3	65
25–40	4	4.3 (3.8, 4.7)	2.8	3	141
40–65	4	3.9 (3.4, 4.4)	3.1	4	170
≥ 65	3	2.9 (1.9, 3.8)	3.0	4	40
Age of secondary case					
0–18	4	4.6 (3.7, 5.5)	3.3	5	50
18–25	3	3.9 (3.1, 4.6)	3.1	4	60
25–40	4	4.4 (3.8, 4.9)	3.0	2	121
40–65	4	3.7 (3.3, 4.2)	2.9	3	160
≥ 65	3	3.3 (2.4, 4.2)	3.0	3	42
Restriction level					
Regional restrictions	4	3.9 (3.3, 4.5)	3.1	3.5	111
Stay at home phase: Start of study period (11th April 2020) to 5th May 2020	4	4.1 (3.3, 4.9)	3.3	3	61
National level 3: 6th October 2020 to 15th October 2020 And 1st December 2020 to End of study period (13th December 2020)	4	4.1 (3.6, 4.6)	2.8	4	128
National level 5: 21st October 2020 to 1st December 2020	3	3.9 (3.4, 4.4)	3.0	4	133

### Fitted distributions

**Fig. 2:**
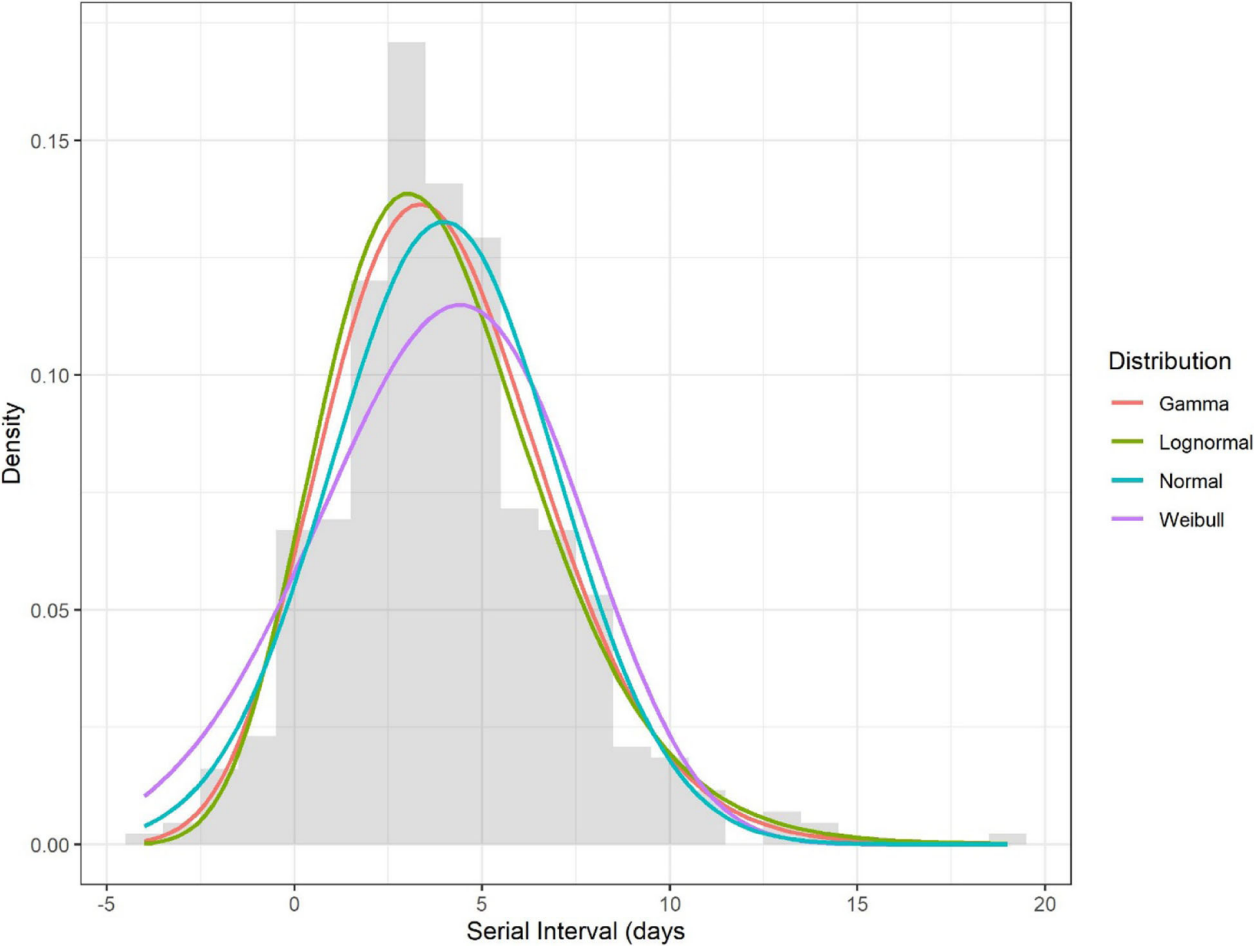
Probability density plots of distributions fitted to the serial intervals of SARS-CoV-2 in the Republic of Ireland. Raw data is shown as a histogram

The parameters of the different distributions fitted to the shifted serial intervals are shown in Table 2. Based on the lowest AIC, the shifted distribution of serial intervals (k = 10) was best approximated by either a gamma distribution with parameters shape = 21.96 and rate = 1.57 (AIC = 2166) or a lognormal distribution with parameters 2.61 and 0.22 (AIC = 2168). Subtracting 10 from the resulting distribution gave 2.5th, 25th, 50th, 75th and 97.5th percentiles of − 1.16, 1.72, 3.60, 5.77 and 10.93 days for the lognormal distribution and − 1.32, 1.84, 3.77, 5.91 and 10.56 for the gamma distribution respectively (Table 3). Figure 2 shows the pdf of each distribution. Table 3 compares the percentiles of the fitted distributions and the raw data.

**Table 2:** Point estimates of the parameters of distribution fitted to the shifted serial intervals (k = 10)

Distribution	Parameter 1	Parameter 2	AIC	ECDF - CDF mean absolute error
Gamma^a^	21.96	1.57	2166	0.027
Lognormal^b^	2.61	0.22	2168	0.027
Normal^c^	13.97	3.01	2186	0.030
Weibull^d^	4.63	15.19	2232	0.038

^a^Parameter 1 = shape, Parameter 2 = rate

^b^Parameter 1 = meanlog, Parameter 2 = sdlog

^c^Parameter 1 = mean, Parameter 2 = standard deviation

^d^Parameter 1 = shape, Parameter 2 = scale

**Table 3:** Comparison of lognormal and gamma distribution percentiles (minus k = 10) with the raw data

Percentile	Raw data	Lognormal distribution	Gamma distribution
0.025	-1	−1.16	−1.24
0.050	0	−0.53	−0.53
0.100	0	0.26	0.32
0.250	2	1.72	1.88
0.500	4	3.60	3.78
0.750	6	5.77	5.87
0.900	8	8.03	7.92
0.950	9	9.53	9.23
0.975	10.2	10.93	10.42

### Sensitivity of estimate to restriction of analysis to cases with one secondary case only

Relaxing the restriction on the number of secondary cases linked to each primary case, from those who infected one other individual resulted in a small decrease in the mean serial interval but did not affect the median serial interval (Table S2, Supplementary Material). Applying no restriction on the number of secondary cases per primary case resulted in a mean of 4.02, whereas restricting to 2 or less, 3 or less or 4 or less, resulted in mean estimates of 3.98, 3.99 and 4.01, respectively. Median serial interval was 4 days, irrespective of the cut-off point used.

### Estimation of presymptomatic transmission

By simulating from the resulting lognormal serial interval distribution and from a previous meta-analysis of international literature on the incubation period of SARS-CoV-2 [10], we found that 67% of draws from our serial interval distribution had greater than 0.50 probability of pre-symptomatic transmission. Using the gamma distribution to simulate the serial intervals, 66% of draws from our serial interval distribution had greater than 0.50 probability of pre-symptomatic transmission.

## Discussion

The reproduction number is an important metric used to describe the efficiency of transmission of a given transmissible disease [17]. The serial interval is an important value used in the estimation of the reproduction number from case data. Based on the national contact tracing data, we estimated the median serial interval of SARS-CoV-2 in Ireland to be 4 days (IQR: 4 days), whilst the mean serial interval was 4.03 (95% confidence intervals 3.75, 4.31). Four percent of our serial intervals were less than zero. These local estimates of the serial interval are important in order to more accurately estimate Rt in Ireland.

The estimate from the current study is within the lower range of earlier international estimates (3.0 to 7.6 days) for serial interval, as reported by Griffin et al. [9]. This lower estimate for Ireland may reflect the timing of the earlier international estimates, with many being taken from earlier stages of the pandemic. Since then, there have been significant international and national efforts to inform those with clinical signs to isolate from susceptible individuals. It is anticipated that with greater awareness and efforts to isolate with the onset of symptoms, pre-symptomatic transmission would account for a greater proportion of all transmission events. In support of this, simulating from the resulting fitted distributions, we estimate that 67% of transmission events in our population occur prior to the onset of symptoms in the primary case. In addition, national public health interventions are likely to have impacted on this value. Previously, it has been shown that national interventions can be expected to reduce the serial interval as within household transmission becomes relatively more important if strict national interventions reduce virus spread elsewhere [12]. An additional possibility could be related to a higher frequency of contacts for the Irish population. However, Ireland has a lower population density than most other countries for which the serial interval has been calculated, suggesting that this hypothesis is less likely.

We found that serial intervals were shorter when either the primary case or the secondary case was older than 64 years of age. To our knowledge, this finding has not been reported before. One potential explanation could be that the incubation period in elderly patients might be shorter, thereby reducing the serial interval when these individuals are the secondary case. However, this hypothesis is not supported by the literature on incubation period, in which there is some evidence to suggest that the incubation period could be longer in these individuals [18]. Similarly, the decrease in serial interval observed when the primary case was an elderly patient could potentially be explained by a reduced latent period, that is the time from infection to the onset of viral shedding, in this cohort. A reduced latent period would mean that elderly patients could have an earlier onset of infectiousness. Alternatively, elderly patients could be more infectious in the pre-symptomatic phase. Interestingly, a recent US study of residents of a skilled nursing home showed high levels of viral shedding in nursing home residents in the pre-symptomatic stage of infection [19].

However, whilst differences to the incubation period, latent period and pre-symptomatic infectiousness in this older cohort might explain this observation, we consider that perhaps more likely is that transmission to these patients is even more biased towards presymptomatic transmission than the rest of the population. For example, given the occurrence of clinical signs in middle-aged (40–64 year-old) patients, it is likely that some mixing within that household (which might include a cohabitant of the same age cohort and/or children) is inevitable irrespective of within-household isolation efforts that are adopted. In contrast, greater efforts to restrict any contact with individuals in the older age cohort may be expected when compared to contacts within younger age groups due to the concern of higher risk of clinical severity in these older age-groups. Consequently, there is less ‘opportunity’ for post symptom onset transmission to older age-groups, such that a greater proportion of transmissions occur pre-symptomatically. In addition, it should be noted that contacts tend to occur with greater frequency within the same age cohort [13]. Therefore, an observation of decreased serial interval according to the age of the secondary case could be confounded by an effect of the age of the primary case, and vice versa. However, it was not possible to determine which of these was the case with our dataset given the lower number of observations in the older age cohort.

We found that the serial interval was similar in Dublin versus the rest of the country. Differences between these two areas might have been anticipated given that one might expect numbers of contacts to be higher in an urban setting versus a largely rural setting, and since at specific time points restrictions have been introduced at a regional level in Ireland [13]. However, we have previously shown that despite this expectation, the number of close contacts reported per for all cases in Dublin was equal to that in the rest of the country [13]. Taken together, neither of these studies support a hypothesis that transmission dynamics might be different in Dublin versus the rest of the country.

Finally, unlike Ali et al. [12], we did not find a difference in serial interval according to the level of restriction. There are several possible reasons for this. Firstly, Ali investigated the impact of restrictions over a relatively short time period (677 pairs over a 36-day time period) during which key restrictions were enforced. However, our study transmission pairs spanned a longer period of time, during which relatively high levels of restrictions were in place. It is therefore possible that we had insufficient data during periods of relaxed restrictions to demonstrate differences in according to restriction level. In addition, at specific time points, the age profile of cases in Ireland differed. It is therefore possible that any changes in serial interval according to restriction level could have been masked by differences in the age profile of cases at that specific time point. In this case we did not have sufficient data to disentangle these effects.

### Limitations

Previous studies investigating the serial interval of SARS-CoV-2 have largely been based on early reports from Asia during the early stages of the pandemic [9]. In those studies, serial intervals were frequently estimated from detailed descriptions of specific outbreaks, tracing forwards and backwords from a smaller number of primary cases in order to trace in detail the particular outbreak [9]. In our study, contact tracing data were collected by trained individuals for the primary purpose of identifying close contacts of infected cases that were at a high risk of onward transmission, not for the purpose of epidemiological parameter estimation. The data are therefore largely collected as part of a trace forward effort. Consequently, there is a risk of mis-specifying the infector within our dataset, as illustrated in the Supplementary Material. To address this, we took additional steps to restrict the analysis to a subgroup of transmission events where there was greater certainty over the identity of the infector. One such step included restricting the analysis to events where the primary case was deemed to have acquired infection via community transmission (where the source of the infection could not be ascertained) whilst the secondary case was deemed to have been infected through close contact with a confirmed case. However, the effectiveness of this approach is dependent on the reliability of the conclusion of the contact tracer in relation to the source of infection. Further diagnostics possibly including viral genome sequencing could be used as an aid to more accurately identify the infector, however these data were not available in our study.

A number of additional limitations are also present in our data. The date of symptom onset was collected during the contact tracing process. Whilst the date of symptoms onset was collected at multiple time points, it is possible that longer serial intervals may have been missed if patients were in the pre-symptomatic phase during the process of testing and contact tracing. This could have biased our estimate downwards. Furthermore, the date of symptom onset is prone to recall bias. To address this, we restricted our analysis to transmission pairs where case details were collected within 1 week of the onset symptoms for both the primary and secondary cases. However, it is still possible that the date of symptom onset is not accurately recorded. Finally, since all reports of serial interval (and other parameters such as incubation period) are based on transmission events where there is a higher degree of certainty about the identity of the infector, it is likely that these reports are based on more intense contacts. This limitation is also present in our study and may have resulted in some bias in our final estimate.

## Conclusions

Based on contact tracing data, we estimate the mean serial interval of SARS-CoV-2 in the Republic of Ireland at 4.03 days. Simulating from serial interval and from an incubation period distribution based on the international literature, we estimate that 67% of transmission events had a greater than 50% probability of occurring prior to the onset of symptoms of the infector.

## Supplementary Information

**Additional file 1: Figure S1.** Secondary or tertiary case? Potential to mis-specify the infector when the primary case results in multiple secondary cases (left). The green circle represents the primary case, the blue circle a contact who did not become a case, and orange circles represent contacts who subsequently became cases. Incorrectly identifying two secondary cases as a secondary and tertiary case (right; that is, an intermediate step infector in a close co-contact) would result in a biased estimate of the serial interval. **Figure S2.** Secondary case or common source? Potential to mis-specify the infector when both the primary case and the case in a recorded contact are acquired from a common unidentified source (ie community transmission). The green circle represents the primary case, the blue circle a contact who did not become a case, and orange circles represent contacts of a case who subsequently became cases. **Figure S3.** Relationship between serial interval, incubation period and time of infection relative to symptom onset of the infector. Symptom onset is indicated with an ‘X’. **Table S1.** Impact of each data cleaning step on number of records. **Table S2.** Impact of restricting data according to the number of secondary cases per primary case.

## Data Availability

The data that support the findings of this study are available from the Irish Central Statistics Office but restrictions apply to the availability of these data, which were accessed by the primary author for the purpose of these analyses and under Section 20(b) of the Statistics Act, 1993 for the purpose of using data collected during the COVID-19 pandemic to aid in the national response.

## Abbreviations

Rt: Time-varying reproduction number
HSE: Health Service Executive
CSO: Central Statistics Office
AIC: Akaike Information Criterion
