# Supplementary material for: Estimation of the serial interval and proportion of pre-symptomatic transmission events of COVID− 19 in Ireland using contact tracing data
Source: BMC Public Health. 2021 Apr 27;21:805. doi: 10.1186/s12889-021-10868-9 (PMC8076671; doi:10.1186/s12889-021-10868-9)
Supplement: Supplementary file 1 — Additional file 1: Figure S1. Secondary or tertiary case? Potential to mis-specify the infector when the primary case results in multiple secondary cases (left). The green circle represents the primary case, the blue circle a contact who did not become a case, and orange circles represent contacts who subsequently became cases. Incorrectly identifying two secondary cases as a secondary and tertiary case (right; that is, an intermediate step infector in a close co-contact) would result in a biased estimate of the serial interval. Figure S2. Secondary case or common source? Potential to mis-specify the infector when both the primary case and the case in a recorded contact are acquired from a common unidentified source (ie community transmission). The green circle represents the primary case, the blue circle a contact who did not become a case, and orange circles represent contacts of a case who subsequently became cases. Figure S3. Relationship between serial interval, incubation period and time of infection relative to symptom onset of the infector. Symptom onset is indicated with an ‘X’. Table S1. Impact of each data cleaning step on number of records. Table S2. Impact of restricting data according to the number of secondary cases per primary case. [file 12889_2021_10868_MOESM1_ESM.docx]

**TITLE PAGE**

**Manuscript title**

Estimation of the serial interval and proportion of pre-symptomatic transmission events of COVID-19 in Ireland using contact tracing data

**Author list**

Conor G. McAloon^1*^, Patrick Wall^2^, John Griffin^3^, Miriam Casey^4^, Ann Barber^4^, Mary Codd^2^, Eamonn Gormley^1^, Francis Butler^5^, Locksley L. McV Messam^1^, Cathal Walsh^6^, Conor Teljeur^7^, Breda Smyth^8^, Philip Nolan^9^, Martin J. Green^10^, Luke O’Grady^1,10^, Kieran Culhane^11^, Claire Buckley^12,13^, Ciara Carroll^12^, Sarah Doyle^12^, Jennifer Martin^12^, Simon J. More^1,4^

**Author affiliations**

^1^School of Veterinary Medicine, University College Dublin, Belfield, Dublin 4, Ireland

^2^School of Public Health, Physiotherapy and Sports Science, University College Dublin, Belfield, Dublin 4, Ireland

^3^Straffan, Kildare, Ireland

^4^Centre for Veterinary Epidemiology and Risk Analysis, School of Veterinary Medicine, University College Dublin, Belfield, Dublin, Ireland

^5^School of Biosystems and Food Engineering, University College Dublin, Belfield, Dublin 4, Ireland

^6^Department of Mathematics and Statistics, University of Limerick, Ireland

^7^Health Information and Quality Authority, George’s Court, Dublin 7, Ireland

^8^Department of Public Health, Health Service Executive West, Galway

^9^National University of Ireland Maynooth, Kildare, Ireland

^10^School of Veterinary Medicine and Science, University of Nottingham, Nottingham, UK

^11^Central Statistics Office, Ardee road, Rathmines, Dublin, Ireland

^12^COVID-19 Contact Management Programme, Health Service Executive, Ireland

^13^School of Public Health, University College Cork, Ireland

***Corresponding author:**

Conor McAloon,

[conor.mcaloon@ucd.ie](mailto:conor.mcaloon@ucd.ie),

01 716 6083

**Supplementary Material 1**

**Figure S1**

**Secondary or tertiary case?** Potential to mis-specify the infector when the primary case results in multiple secondary cases (left). The green circle represents the primary case, the blue circle a contact who did not become a case, and orange circles represent contacts who subsequently became cases. Incorrectly identifying two secondary cases as a secondary and tertiary case (right; that is, an intermediate step infector in a close co-contact) would result in a biased estimate of the serial interval.


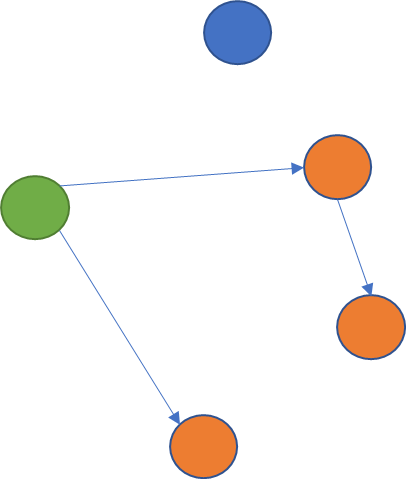


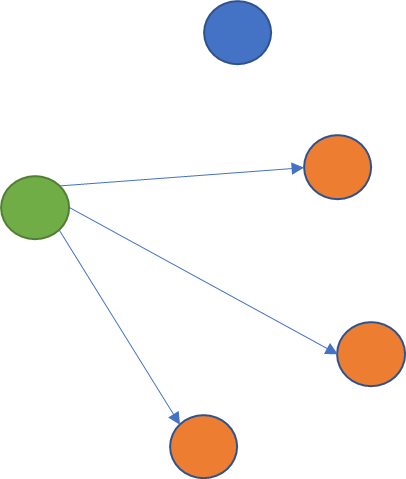


**Figure S2**

**Secondary case or common source?** Potential to mis-specify the infector when both the primary case and the case in a recorded contact are acquired from a common unidentified source (ie community transmission). The green circle represents the primary case, the blue circle a contact who did not become a case, and orange circles represent contacts of a case who subsequently became cases.


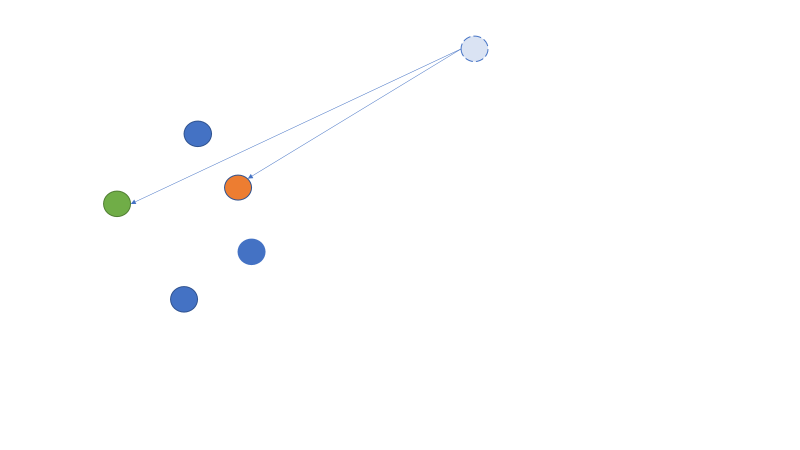


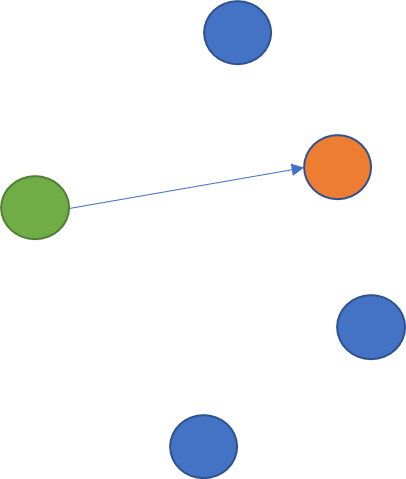


**Figure S3** Relationship between serial interval, incubation period and time of infection relative to symptom onset of the infector. Symptom onset is indicated with an ‘X’.

**
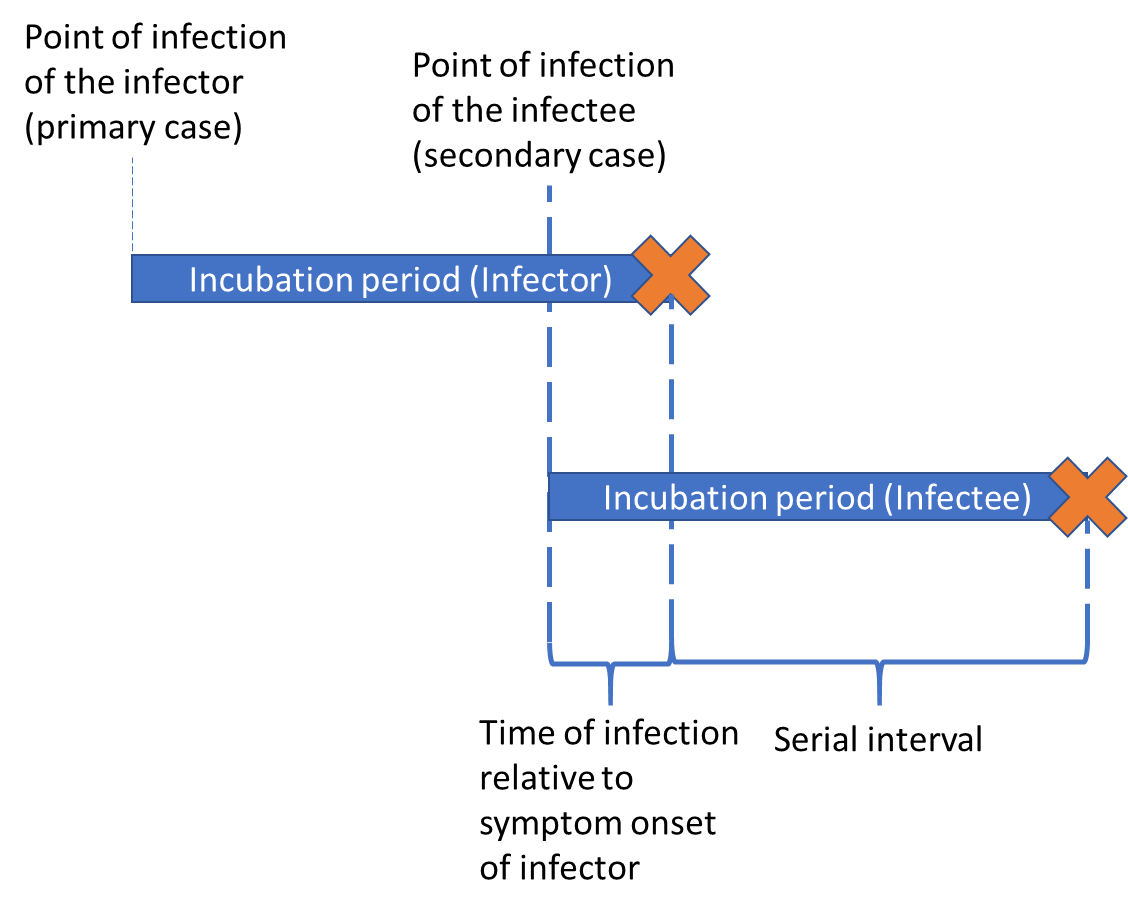

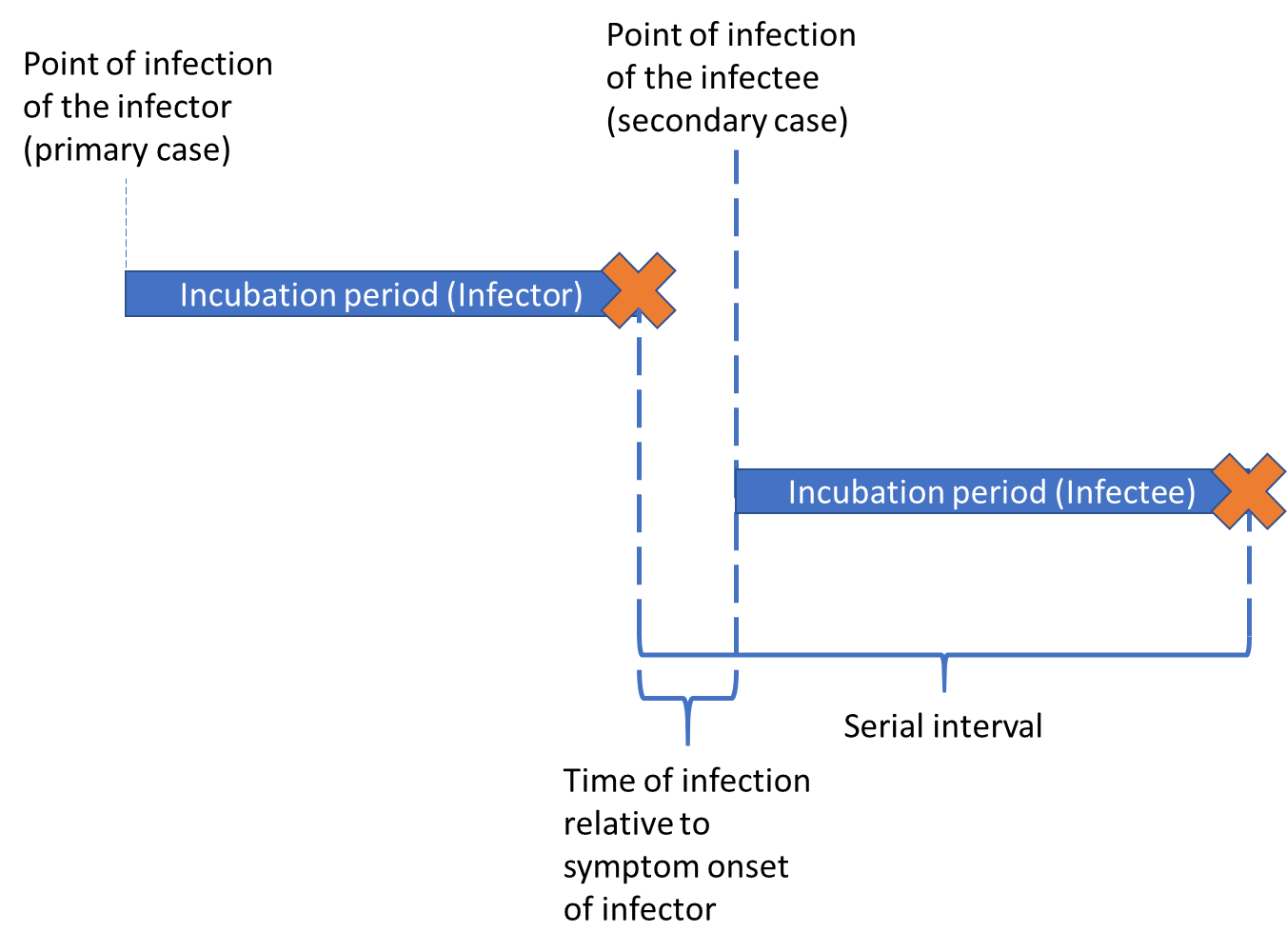
**

**Table S1** Impact of each data cleaning step on number of records

| **Step** | **Number of unique cases** | **Number of unique contacts** |
| --- | --- | --- |
| Initial data read in | 111,251 | 293,597 |
| Restrict to contacts who subsequently became cases | 27,656 | 41,762 |
| Remove cases who have  contacts appearing more than once | 22,030 | 36,650 |
| Primary case attributed to community transmission, secondary case to close contact with confirmed case | 6,280 | 10,475 |
| Both the primary and secondary cases were symptomatic and had known symptom onset date. | 3,551 | 5,206 |
| Remove data within 30 days of last entry | 2,073 | 2,968 |
| Restrict to close contacts | 2,068 | 2,961 |
| Filtering to serial intervals between -10 and 28 days in length | 1,877 | 2,693 |
| Filter to those where date of symptom onset was within 7 days of date of contact tracing phone call  Used as sensitivity analysis dataset – no restriction on number of secondary cases | 1,406 | 1,999 |
| Restrict to individuals who infected only one other person | 433 | 433 |

/

**Table S2** Impact of restricting data according to the number of secondary cases per primary case.

| **Cut off point (number of secondary cases per primary case)** | **Number of primary cases** | **Median serial interval** | **Mean serial interval** |
| --- | --- | --- | --- |
| 2 | 818 | 4 | 3.99 |
| 3 | 1074 | 4 | 3.98 |
| 4 | 1225 | 4 | 4.01 |
| 5 | 1301 | 4 | 4.03 |
| 6 | 1349 | 4 | 4.02 |
| 7 | 1368 | 4 | 4.03 |
| 8 | 1380 | 4 | 4.02 |
| 9 | 1391 | 4 | 4.02 |
| 10 | 1398 | 4 | 4.02 |
| 11 | 1402 | 4 | 4.02 |
| 12 | 1405 | 4 | 4.02 |
| 13 | 1406 | 4 | 4.02 |
